# To the Editors of the Medical and Physical Journal

**Published:** 1820-03

**Authors:** A. P. W. Philip

**Affiliations:** Worcester


### *To the Editors of the Medical and Physical Journal.*

GENTLEMEN,*

I beg that you will have the goodness to insert the inclosed letters and observations in the Medical and Physical Journal for next month.

I have the honour to be,
Gentlemen,
Your obedient humble Servant,

*Worcester, February* 14, 1820. A. P. W. PHILIP.

---

### *Dr. W. Philip's Reply to Mr. Brodie, Dr. C. H. Parry, and others.*

Dr. Cooke, in the introduction to his work on Nervous Diseases, relates several experiments performed by Mr. Brodie; the results of which, Mr. Brodie thinks inconsistent with certain inferences in my Inquiry into the laws of the vital functions. Dr. Cooke also quotes, from the Quarterly Journal for April last, the observations of three members of the Royal Society, who think that I was deceived in the result of certain experiments related in the above Inquiry, but without noticing my reply to these observations in the following number of the same journal. I addressed a letter to the gentleman, complaining of this circumstance, to which he returned an obliging answer, with permission to make it public. He says, "The Quarterly Journal for April last was sent to me by a friend, who thought that the experiments related in it were connected with the subject of my Introduction. I have seen no other number of that journal, and was not aware that you had made a reply; otherwise I should have thought it a duty to have detailed that reply. If my book should go to a second edition, which I think not improbable, I shall be happy to supply the deficiencies of the first.

The Introduction to Dr. Cooke's work also induced me to address the following letter to Mr. Brodie.

SIR, *Worcester, January* 25*th*, 1820.

A gentleman from London informed me, that he heard it publicly mentioned, that you are one of the gentlemen who gave the statement in the Quarterly Journal of April last, in answer to some observations in the Appendix to the second edition of my Inquiry into the Laws of the Vital Functions. This report appears to be confirmed by what is said in the 129th and following pages of Dr. Cooke's late Treatise on Nervous Diseases. It is with considerable surprise that I have there seen the experiment which was detailed in the above-mentioned statement in the Quarterly Journal again brought forward, as affording a refutation of the results of some of my experiments, after I have repeatedly pointed out, that the circumstances of that experiment are in *no essential respect similar* to those of the experiments in question; and it is universally admitted, that, in such experiments, even *the slightest deviation* may influence the result. The only thing which can excuse Dr. Cooke's quoting from the Quarterly Journal the experiments and observations of my opponents, and wholly passing over in silence my reply to them, is, that he may not have seen the following number of that journal, which contains my reply.

I ask you, sir, as a gentleman and a man of science, to say, whether the above-mentioned experiment can be regarded as a fair repetition of my galvanic experiments?

---

* The printing of the Journal had extended beyond the *first department*, before this paper of Dr. Wilson Philip was received by us. We have therefore been obliged to insert it here, instead of placing it amongst the *Original Communications*. EDIT.

I am mortified to find, that I have made myself so ill understood by you in my Inquiry into the Laws of the Vital Functions, that you have in Dr. Cooke's work brought forward several experiments with a view to refute certain parts of that Inquiry, which do not, as far as I can judge, at all interfere with any opinions maintained in it.

In the first of these experiments, the nerves sent by the eighth pair to the cardiac portion of the stomach were divided, and, at the end of a week and three days, digestion was found to be going on as usual. This experiment was related to me by Sir Everard Home, in a letter which I still have by me, as performed by Majendie more than a year before the publication of the first edition of my Inquiry, in the 168th page of which it is mentioned; and I cannot perceive any reason why a different result should have been expected from it. It is shewn in my Inquiry, that, if any considerable part of the influence of the brain or spinal marrow be withdrawn from the stomach and lungs, the secretions of these organs are deranged. The par vagum evidently conveys to the great chain of ganglions the influence of the brain. When it is divided at a short distance from its origin, the influence it conveys is cut off; but I cannot perceive how this can at all be done by dividing the particular branch of this nerve, which goes to the cardiac portion of the stomach. Before it arrives at this place, it has formed innumerable connexions with the great sympathetic and ganglions. They have received from it the influence of the brain; and, if nerves, going to any particular organ, be divided, there are every-where, such are the precautions of nature, numerous anastomosing branches still capable of conveying the necessary influence, as long as it is duly supplied from its sources.

Had you, sir, been more conversant with the effects of dividing the par vagum, you would not, I think, have made the observations quoted in Cooke's work relating to the influence of the state of the lungs on the stomach after the division of these nerves in the neck. The dyspnœa is at first so slight as often to be hardly perceptible; sometimes, indeed, it is not at all so. It only becomes considerable, as phlegm accumulates in the lungs; yet, if the animal be allowed to eat as soon as the nerves are divided, efforts to vomit immediately ensue. In one case, in which Dr. Hastings had divided them, and the animal was immediately allowed to eat, the food it had taken, in consequence of the efforts to vomit, accidentally got into the trachea in such quantity as to occasion suffocation, and the animal died within five minutes after the division of the nerves, and without any dyspnœa previous to the act of suffocation having been observed. If you will take the trouble to re-peruse the account of my galvanic experiments, you will find, that the digestion was sometimes most impaired when the dyspnœa was least so, and *vice versa*.

The observations just made respecting the division of the cardiac nerves apply, if possible, with greater force to the experiments in which the great nerves of the leg were divided. Dr. Monro, I believe, relates similar experiments in his Lectures, but without drawing your inferences from them. It is well known, that nerves accompany the vessels throughout every part of the body in such number, that, could we destroy all other parts of a limb, leaving the nerves alone, they would still give the appearance of the entire limb. Now, it is shewn by experiments related in the above Inquiry, that the nerves which supply the vessels belong to the ganglian system,—an inference well supported by the observations of the anatomist; and that it is on these nerves, and not on the nerves immediately derived from the brain and spinal marrow, that secretion depends. The use of the nerves you divided, is to convey impressions to and from the limb, and to excite the muscles of voluntary motion. It is true, they receive twigs from the ganglian nerves, as all parts do, but the division of these can be of little consequence, as the number of ganglian nerves and their anastomoses may be said to be almost without end. It is the character of the ganglian nerves that they every-where anastomose, and that each may convey the influence of many, while the nerves arising directly from the brain and spinal marrow, convey the influence only of that part of those organs from which they take their rise. These facts are illustrated by many experiments related in my Inquiry. It might as well be said, that the blood supplied by any of the great arteries of a limb is not essential to the health of the limb, because, when the artery is secured by ligature, the anastomosing branches can perform its functions: as that the division of any ganglian nerve not impairing the function of secretion proves, that the influence it conveyed is not necessary to the due performance of that function. While the source of nervous influence is entire, nature has provided many channels for that part of it on which life depends.

The next experiment related in Dr. Cooke's work is one in which the source of nervous influence was lessened, and there were evident marks of deranged secretion in the wound. It is true, that you observe, that " the circumstance of the union being incomplete, may be reasonably attributed to the animal being in a torpid state, and remaining apparently without nourishment for many months." But all who have been much in the habit of experimenting on frogs know, that their health is not readily influenced by causes which greatly impair that of warm-blooded animals. Granting, however, that the causes you state did to a certain degree operate, will it be admitted, that, in estimating those which prevented the proper healing of the wound, nothing is to be ascribed to such a privation as that of the lower part of the spinal marrow? The effect was such as corresponds with the results of my experiments. Even in those in which the greatest part of the spinal marrow was destroyed, which could be done without immediately destroying life, the stomach was not more affected than by dividing one of the eighth pair of nerves; and, when the whole of the lumbar portion was destroyed, some degree of digestion still went on even in the warm-blooded animal, which is so much less tenacious of the living powers than that of cold blood. It appears from many of the experiments of M. le Gallois, as well as of my own, that the lower part of the spinal marrow is the least essential of those parts which prepare the nervous influence.

The inference to which your view of the experiments which Dr. Cooke quotes from you have led you, that only some of the secretions are under the influence of the nervous power, (Dr. Cooke's work, page 134,) seems unphilosophical, and would therefore, on this account alone, have appeared highly improbable.

When I consider the nature of this letter, sir, I need not, I trust, beg of you to favour me with as early a reply to it as your engagements will admit of.

I have the honour to be, sir, your very obedient humble servant,

A. P. W. PHILIP.

*To B. C. Brodie, Esq.*

The following is the Answer which I have received from Mr. Brodie.

Sir, 16, *Saville Row; Feb.* 4, 1820.

You were correctly informed that I was one of the three Fellows of the Royal Society who were formerly requested to undertake the repetition of your experiment on the application of the galvanic influence to the stomach after the division of the nerves of the eighth pair. After reading your observations on our experiment, I can discover only one point of difference between it and the original experiment made by yourself, namely, that in ours, the galvanic influence was applied by a succession of contacts instead of a continual stream. How far this may have affected the results I will not pretend to determine; but, as you suppose that it might have done so, I have (since I had the honour of receiving your letter of the 25th ult.) made the experiment again, taking care that it should very accurately resemble your's in this and in all other respects. The rabbit was fed with parsley; and, immediately after the nerves of the eighth pair were divided, he was subjected to the influence of the galvanic battery, and this was continued for upwards of seven hours, at the end of which time the contents of the stomach were examined, and found to have precisely the same characters, the same odour, appearance, and consistence, as in another rabbit which had been fed in the same manner, and in which the nerves had been divided at the same time, but on which the galvanic battery had not been employed.

You may be assured, that neither my friends nor myself have the smallest desire to depreciate the value of your labours; and I hope that I have sufficient zeal in the cause of science to have experienced a real pleasure, if I could have met with results similar to those obtained by yourself. That I have not done so is not to be considered as my fault, and ought not to be regarded by you as a matter of offence. If the subject be worthy of further investigation, I doubt not that some competent experimenter will sooner or later undertake to prosecute it: for myself, I take the liberty of declining to enter into any controversial discussion, for which I have neither leisure nor inclination.

I beg to offer to you my apologies for not having acknowledged your letter sooner; the delay has arisen from my desire to make a second repetition of your experiment previous to my writing to you.

I have the honour to be, sir, your obedient servant,

B. C. BRODIE.

*To Dr. A. P. W. Philip, Worcester.*

In reply to Mr. Brodie's Letter I wrote the following, inclosing the declarations of Dr. Hastings and Mr. Sheppard, which I am about to lay before the reader.

Sir, *Worcester; Feb.* 7, 1820.

I was yesterday favoured with your reply to my last. You will be troubled with another letter, which I transmitted through an uncle who resides in London, and which left Worcester before your letter reached me. I hope you will believe that it is impossible for me to associate any degree of unfairness with the opinion I have always entertained of you, but the experiment in question has been so often repeated, and with such circumspection, and that by different people, that there are no physiological facts of which I am better assured, than that a certain power of galvanism will remove dyspnœa, and restore to the stomach the power of altering the food, after the eighth pair of nerves are divided in the neck. One man may be deceived; but that the number who have witnessed this experiment, and that repeatedly, should have been so, is impossible. I am therefore persuaded that the circumstances of your experiment and mine have differed in some essential respects, although you are not at present aware of it.

I can form no judgment either of the circumstances of your experiment, or the precise result, till you have answered the following questions, which you will particularly oblige me if you will have the goodness to do, on receipt of this letter.

1. Was the animal fed after a fast of some continuance immediately before the experiments?

2. Was the twitching of the fore legs constant during your experiment?

3. Was the dyspnœa occasioned by the division of the nerves relieved by the galvanism?

4. Did vomiting come on in the galvanised rabbit?

5. What was the appearance of the food in the stomach, as exactly as it can be stated?

You do not, I hope, sir, think me so illiberal, not to say irrational, as to be offended at any one for observing different results from any of my experiments; but you will allow that, repeating an experiment made by me, without due attention to what I consider its most essential circumstances, and laying the repetition before the Royal Society, and thus influencing many of its members against me, without the subject having been mentioned to me, and consequently without an opportunity of vindicating myself, having been given to me, are just causes of complaint.*

I can truly say, I believe that I have as little leisure or inclination for controversial discussion as you have, but in one particular I differ from you. It is certainly optional with every man whether he will enter into discussions or not, but when he may retire from them, is not equally so. I can feel no wish to retire from the discussion in question till it is finally settled; and, if we cannot otherwise discover the circumstance which has caused so great a difference in the result of our experiments, I shall be happy (as I shall have occasion to be in London next autumn, if not sooner), to repeat my experiment in your presence and that of some other of the most eminent men of our profession, and their report will determine the question.

I send on the other side of this paper the declaration of Dr. Hastings and Mr. Sheppard, both men of the highest respectability, the former well known to Dr. Burrows, Dr. James Johnson, and others of our profession in London, the other, to Mr. Cline, Mr. Astley Cooper, and others.†

I have the honour to be, sir, your faithful humble servant,

(Signed) A. P. W. PHILIP.

*To B. C. Brodie, Esq.*

THE INCLOSED DECLARATIONS.

I hereby declare, that, having very often divided the eighth pair of nerves in the neck of the rabbit, and always found that it immediately put a stop to digestion, the food eaten previously to the division of the nerves being found in the stomach unchanged, however long the animal lived after the division of the nerves, I was requested by Dr. Philip to make the following experiment. Having allowed a full-grown rabbit to eat as much parsley as it chose, after a long fast, and shaved the hair off its stomach, I divided the eighth pair of nerves in its neck, and coated the lower part of the nerves with tin-foil, and bound a shilling over the stomach. It was then exposed to the galvanic influence, by connecting the tin-foil and shilling with the opposite ends of a galvanic trough, of such power as to keep up a constant twitching in the fore-legs. The difficulty of breathing which always succeeds the division of the eighth pair of nerves had come on before the galvanic apparatus was arranged. Upon the application of the galvanism the breathing soon became free. When the power of the trough failed, or we intentionally discontinued the application of the galvanism, which we did repeatedly, the breathing always became oppressed, but was again rendered free on the re-application of the galvanism. This continued to be the case for twelve hours, after which the breathing could not be wholly relieved by the galvanism, inflammation of the lungs having supervened, and during the last hour of the animal's life the dyspnœa was

---

* I might also have stated in the above letter, that the circumstance of the names of all the gentlemen who made the experiment having till now, a space of several years, remained unknown to me, greatly increased the difficulty of proving the truth of my experiment.

† I made no reply to Mr. Brodie's observation, that he could discover only one point of difference between the experiment detailed by himself and his friends in the Quarterly Journal, and mine, because I could only repeat what I had said in the following number of that Journal. If Mr. Brodie had had occasion to repeat the experiment frequently, he would have seen, that all the circumstances I there mention are capable of influencing the result. It is needless, however, to insist upon this, as he acknowledges that there was a deviation from my method, which might have influenced the result. But I am assured that he will not obtain a correct result, till all the circumstances I mentioned, and particularly the proper strength of the galvanic power, as well as its mode of application, is attended to.

very great. No attempt to vomit occurred in the animal except once, when the galvanic influence had become very weak, ten or twelve hours after the division of the nerves; whereas, in every instance in which I have divided these nerves without the application of galvanism, and I have done so more than a dozen times in the rabbit, efforts to vomit always soon ensued.

On examining the animal after death, the lungs were found highly inflamed. The contents of the stomach, instead of being such as above described, presented the appearance of those of a healthy stomach, in which digestion had been going on for many hours.

I am ready to attest the truth of every part of the preceding statement in any way in which I may be called upon to do so.

(Signed) CHARLES HASTINGS.

*Worcester; Feb.* 6, 1820.

Three other medical men were present at this experiment, and particularly examined both the stomach and lungs, and expressed themselves perfectly satisfied with the result. C. H.

Having, at the request of Dr. W. Philip, divided the eighth pair of nerves in the neck of two small dogs, which were allowed to eat as much lean raw mutton, cut into small pieces, as they chose immediately before the experiment, after having fasted for many hours, I subjected one of them to the galvanic influence, by coating the lower parts of the divided nerves with tinfoil, and connecting it with one end of the galvanic trough, while the other end of the trough was connected with the region of the stomach, which, before the experiment, had been shaved, and a three-shilling piece bound upon it. The power of the galvanism was such as to occasion a twitching of the forelimbs during the whole experiment. The dog which was not galvanised was immediately seized with dyspnœa and efforts to vomit. In the other, neither was observed in the slightest degree at any period of the experiment, except when for a few seconds the galvanic influence was intentionally discontinued, during which the breathing became very laborious, again becoming free as soon as the galvanism was restored. This dog lived above two hours. The other dog was still alive, though extremely weak, at the end of four hours, at which time it was killed by a blow on the head.

On examining the stomach of the galvanised dog, the mutton was found in a soft, half-dissolved state, all character of muscular fibre having disappeared. The lungs, on examination, were found perfectly healthy, but rather of a florid colour. In the stomach of the other dog the bits of mutton still retained their firmness, and, on being cut into, displayed both the red colour and fibrous appearance of the muscle, which did not seem to be at all diminished. The lungs were found greatly congested, and collapsed very imperfectly, the surface being covered with patches of a dark-red colour.

The above experiment was made in presence of the house surgeon and pupils of the Infirmary, who all examined the state of the stomach and lungs, and expressed themselves satisfied with the result.

The accuracy of the above statement I am ready to attest in any way in which I may be required to do it.

(Signed) JAS. P. SHEPPARD.

*Worcester; Feb.* 7, 1820.

The following is the letter alluded to in the preceding.

Sir, *Worcester; Feb.* 6, 1820.

About ten days ago I wrote to you, in consequence of some observations published in Dr. Cooke's work on Nervous Diseases. You will perceive that the occasion requires that my letter should be published. It will go to the press in a few days. I have requested a gentleman, on whose discretion I

can depend, to deliver this to you, and to learn from you what reply, if any, you wish me to state as having received from you, when I mention my having sent you the above letter.

I have the honour to be, sir, your very obedient humble servant,

(Signed) A. P. W. PHILIP.

*To B. C. Brodie, Esq.*

After waiting till the 10th for Mr. Brodie's reply to these letters, I addressed the following letter to that gentleman, with regret that the advanced period of the month did not allow me to wait a longer time for his reply than the 13th.

SIR, *Worcester, Feb.* 10, 1820.

I am sorry again to trouble you so soon; but, as from the introduction to Dr. Cooke's work and other reasons, I feel it necessary that the letters which I have addressed to you should be laid before the public, and am particularly anxious that your reply should appear in the form most agreeable to you, it is necessary that I should inform you, that my letters, for reasons with which I need not trouble you, must leave this for the press on the 14th inst. before I can hear from you by the mail of that day. As I have not heard from you, in reply either to my letter of the 7th, or to that delivered to you by my uncle on that day, in which I mention the intended publication of my first letter, and beg to know what, if any, reply you wish me to make public. I am obliged to write to-day, that you may not be called upon to reply by return of post; and I am very sorry, and beg to apologize, that circumstances oblige me to solicit so early a reply as I now do, which your numerous professional engagements may render inconvenient to you. If I hear from you, I shall of course follow the directions you give respecting the letter I have received from you. If not, I shall consider your silence as leaving me to the dictates of my own feelings. In that case, I shall act as I should wish another to act towards me under the same circumstances; and give to the public, with my letters, your reply to my first letter; and I shall take care afterwards to make equally public any reply you may favour me with to my other letters, as far as you wish me to do so.

I have the honor to remain, sir, your faithful humble servant,

(Signed) A. P. W. PHILIP.

*To B. C. Brodie, Esq.*

I received the following answer from Mr. Brodie on the 13th.

SIR, *Saville Row; Feb.* 12, 1820.

I beg to acknowledge the receipt of your letters on the 7th and 12th instant, and of Dr. Hasting's and Mr. Sheppard's additions to the former.

In the experiments related in my last letter, the twitchings of the fore-legs were nearly constant. There was no vomiting. It was not observed that the respiration was different from that of the other rabbit which was not galvanised. The food in the stomach had the appearance of masticated parsley.

I shall be glad of the opportunity of seeing you make the experiment whenever you visit London; I undertake to promise that every facility shall be afforded you of doing so. I assure you it will give me much satisfaction to observe such results as you anticipate.

In closing this correspondence, I beg to remark, that you are in an error, if you suppose that I laid the repetition of your experiment before the Royal Society with a view to influence any of its members against you, without your being able to vindicate yourself. I only joined two other gentlemen in repeating the experiment, at the request, and for the satisfaction, of those with whom the management of the concerns of the Royal Society principally rests.

You are at perfect liberty to publish my letters; and I have the honour to be, sir, your obedient humble servant,

(Signed) B. C. BRODIE.

*Dr. W. Philip, Worcester.*

To this Letter I returned the following answer:

SIR, *Worcester; February* 14, 1820.

I am favoured with your letter, and am much obliged by the readiness with which you offer me every facility in the repetition of my experiment. I shall have much pleasure in making it with you; and, from your answer to several of my queries, I have little doubt that we shall detect the cause of the difference of result. You say, in reply to my last query, "the food in the stomach had the appearance and odour of masticated parsley." By this I understand that the parsley was, throughout the stomach, in the state here described. This state is very different from that in which the contents of the stomach were found in your first experiment, (see the Quarterly Journal for April last, page 164;) for which, as I observed in my reply to it, I could not account. You say, in reply to my fourth query, that "there was no vomiting" in the galvanised rabbit; that is, no efforts to vomit, as nothing is ever brought up after the division of the eighth pair of nerves. It appears, from the observations in the 154th and 155th pages of my Inquiry, that we have reason to believe that no means will prevent the efforts to vomit after the division of these nerves, but some degree of digestion going on in the food which lies in contact with the coats of the stomach. You observe, in reply to my second query, that the twitchings of the fore legs were only "nearly constant," and in your experiment the galvanism was only continued for seven hours; while, in the most decisive of my experiments on rabbits, it was continued for sixteen hours. In the result of my last galvanic experiment, in which the stomach was exposed only to a comparatively small degree of galvanic influence, you will observe a striking resemblance to the result of your experiment. It is said, in page 232 of my Inquiry, 2d edition, "The food was but little altered, retaining the colour, smell, and stringiness, of the parsley. It was, however, sufficiently exposed to the galvanic influence to prevent the vomiting." This perfectly corresponds with your account of the state of the contents of the stomach in your last experiment, but is very different from that given of them in your former experiments. In my experiments, the metal was always applied to the lower part of the pit of the stomach: when it is applied near to the end of the sternum, hardly any part of the stomach is in the galvanic circle. You forgot to answer my first query.

It is a circumstance in this discussion, you will allow, which particularly deserves notice, that the proofs on our side are all of a positive kind; your's, necessarily, of a negative kind. We only speak of what we could not have seen, had it not existed; you, of what you were not able to see, which many things, besides what you suppose, might have prevented you seeing. You now admit that, in your first experiment, there was a deviation from my method which might have occasioned a difference of result; yet you were then quite as sure of the contrary with regard to that experiment, as you now are with regard to the present; for you say, in the Quarterly Journal for April last, page 162, "that the experiment might be repeated with the greater accuracy, the paper was put into these gentlemen's hands, *who implicitly followed the directions contained in it.*"

If you will take the trouble to re-peruse my letter, you will find that I do not even surmise that your motive, in any thing you have done, was such as you mention. I merely stated the facts, and their necessary consequence: and now that you are sensible that the experiment was not a correct repetition of mine, you will regret, I am persuaded, that it was presented to the Society.

I have the honour to be, Sir, your faithful humble servant,

*B. C. Brodie, Esq.* A. P. W. PHILIP.

Having occasion to address the public on the subject of the preceding letters, I beg leave to make some farther observations connected with it. I do not feel myself called upon to reply to various observations which have been publicly made on the opinions maintained in my Inquiry into the Laws of the Vital Functions. I feel no anxiety in leaving them to the judgment of those who are versed in the subjects to which they relate; but there are two instances in which my meaning has been so unaccountably misunderstood, and that in works which are in the hands of many members of our profession, that, without some explanation on my part, I must appear to maintain opinions which I believe to be wholly unfounded.

The first of these works which I shall mention, is entitled *Additional Experiments on the Arteries of Warm-blooded Animals*, by Charles Henry Parry, M.D. F.R.S. &c. In the Inquiry above mentioned, I had occasion to reply to some observations of Dr. Parry, father of Dr. C. H. Parry, in his Elements of Pathology and Therapeutics, on the opinion which I had maintained respecting the nature of inflammation. The latter gentleman has given an answer to this reply in the above treatise. It is with reluctance that I notice his answer, because I am wholly at a loss to account for his, I may almost say, uniform misconception of my meaning.

In the 160th page, he begins his answer by observing, "Dr. Wilson Philip, taking up the opinion of Bichat and others, has indeed attempted a direct proof from experiment in favour of the exclusive influence of the capillaries in carrying on the circulation." If Dr. C. H. Parry can point out any passage in my treatise in which such an opinion is expressed, I shall be much more surprised than he can possibly be, that any one should maintain a position so extravagant. Many quotations from my treatise follow, the meaning of all which he appears to me to misapprehend. I would ask Dr. C. H. Parry, where I have maintained that the larger arteries do not possess "an equal and proportional share of power" (page 161) with the capillaries? Where have I made any inference from the irregular motion of the blood, observed under certain circumstances in the capillaries, respecting the cause of circulation? (page 163.) Where have I maintained, that "no influence can be exerted through them," namely, the heart and large vessels, "by stimuli on the capillaries," (page 168.) With respect to the quotation in page 165, if Dr. C. H. Parry will take the trouble to re-peruse it, he will find that I speak of the result of Dr. Parry's experiments, not of any thing which "Dr. Parry has himself asserted." Similar observations apply to other passages.

I must beg the reader's patience while I say a few words in reply to the observations of Dr. C. H. Parry, on the quotation which he gives from my treatise, in the 175th page. He observes, "Dr. W. Philip continues, The truth of this inference appears indeed from direct experiments; for, from those made with a view to ascertain the state of the vessels in an inflamed part, it clearly appears, not only that the velocity of every part of the blood was lessened, which Dr. Parry admits may be the case, supposing the lessened momentum arising from this cause more than compensated by the increased quantity of blood, but that the general momentum of the blood also in the inflamed part was lessened, because the blood was observed to move more and more slowly, till in the most inflamed part it ceased to move altogether."

Dr. C. H. Parry's first observation on the above passage is, "This sentence is certainly not intelligible." It would possibly have been more correct, had he said, "not intelligible to me." "In the first place," Dr. C. H. Parry proceeds, "Dr. Parry has no admission with regard to diminished velocity, which is entirely an assumption of the author, but simply as to the rate of momentum." Dr. Parry observes, in his Elements of Pathology and Therapeutics, "Neither will this conclusion be invalidated, were it even proved, according to the opinion of Dr. Wilson Philip, that the velocity of the blood in the vessels of an inflamed part is diminished, unless it be also proved that the velocity is diminished in a greater proportion than the quantity is increased." This passage I quoted, and only argued on it, to shew that the velocity is diminished more than the quantity is increased.

2

"In the second place," Dr. C. H. Parry proceeds, "when we consider that the momentum is the product of the quantity and velocity, it is not possible to suppose 'a lessened momentum more than compensated by an increased quantity of blood.' If in this passage the term velocity had been substituted for momentum, there would have been some appearance of meaning." The passage here given as a quotation from my treatise, is indeed nonsense; but it is Dr. C. H. Parry's, not mine. The reader will observe, from the quotation in the last paragraph but one, that the passage in my treatise stands thus: "Supposing the lessened momentum arising from this cause," namely, the lessened velocity, "more than compensated by the increased quantity of blood." This to most men, I believe, would be perfectly intelligible.

In the 174th page, Dr. C. H. Parry also uses very strong expressions, under similar circumstances. He observes of me, "In combatting Dr. Parry's opinions on these subjects, he thus expresses himself: 'Does it not seem a necessary inference, that the blood is moved in the capillaries by the power of these vessels themselves, and consequently that, if they are debilitated, the momentum of the whole part, as well as its velocity, must be less than in health.' Now, though this is tautological and superfluous, as relating to a case where the quantity of matter is allowed to be increased, because there is no instance in which the momentum is diminished, where the quantity is increased, without such a diminution of velocity, &c." If Dr. C. H. Parry will take the trouble to refer to my treatise, he will find that he has again misquoted it. He, indeed, makes the sentence such as deserves even more than the severity with which he treats it.

I shall trouble the reader with but one more passage from Dr. C. H. Parry's treatise (page 177): "With regard to some of Dr. Wilson Philip's experiments, I must confess that I have repeated them with very different results. To avoid the cruel, because sometimes uncertain, process of crushing the brain with the blow of a hammer, and to remove altogether the injurious effects of the violent motion which it may occasion, I have inclosed the whole head in a pair of wide and flat-mouthed pincers with large handles, forming a powerful lever. The whole head was thus, without the least disturbance or chance of an imperfect effect, crushed in the smallest possible space of time. The result of this sudden and certain death has not been a suspension of the capillary action, but its increase." Dr. C. H. Parry must be aware that, when the brain is crushed by the blow of a hammer, which can never be uncertain if the hammer is very large, its destruction is instantaneous. It occupies but a small fraction of the time which must be employed in crushing it by the means which Dr. C. H. Parry employed; on this account, much more cruel. His remark, therefore, that it was crushed in the smallest possible space of time, cannot be admitted. The effect in his experiment was the same as that which I have mentioned, as ensuing when the brain is imperfectly crushed, (see my Treatise, page 94, 2d edition.) If Dr. C. H. Parry will take the trouble to look into it, he will see how much the result of such experiments depends on the suddenness with which the brain is crushed. Is it fair, in repeating physiological experiments, to deviate from the method of the original experimenter in the most essential respects, and then adduce the necessary difference of result, as a proof of his inaccuracy?

I regret that I have been obliged to reply to a son of Dr. Parry in the way in which I have now done. Dr. C. H. Parry cannot find a better example than that of such a father. He will see in his writings nothing that had not been duly considered; and his replies to those who dissented from him, are remarkable for the modesty of a philosopher, and the language of a scholar and a gentleman.

The other work above referred to, is the London Medical and Physical Journal. In a Proemium to the 43d volume of this work, by Mr. Hutchinson, just published, in which this gentleman has taken upon himself the duty of instructing the public respecting "the progress of medicine and its auxiliary sciences," there are many observations relating to my Inquiry into the Laws of the Vital Functions. In the 20th page he observes of me, "He also asserts that he has refuted the notions of Bichat respecting the functions of the ganglionic system of nerves. Although the positive argument of the life and growth of the fetus without brain and spinal marrow may alone be considered a sufficient refutation of the negative arguments, formed for the most part on loose and remote analogies, which Dr. Philip advances against the doctrines of Bichat." What is meant by my assertion that I have refuted the notions of Bichat respecting the ganglian system, and the loose and remote analogies, I know not, unless they allude to my referring the reader to certain experiments detailed in my Inquiry, the results of which are inconsistent with his opinions on this subject. In a note referred to in what Mr. Hutchinson says respecting the fetus without brain and spinal marrow, it is observed, "Dr. Philip does not even notice this fact, although there are instances of it recorded by Morgagni," &c. Now, I have not only mentioned this fact repeatedly, but have entered into a disquisition of some length respecting it, in both editions of my Inquiry;* and, in the second edition, described a case of this kind which I had an opportunity of examining.†

Mr. Hutchinson continues: "The only important argument brought against the doctrine of Bichat, is, that the functions he stated to depend on the influence of the ganglionic system of nerves, cease on the destruction of the brain and spinal marrow." (page 21.) This gentleman here passes unnoticed various other parts of my treatise: all the experiments, for example, which I have detailed for the purpose of proving that the organs which are under the ganglian system are affected by stimuli applied to the brain and spinal marrow. He does not seem aware that I had ever made such experiments.

A little lower in the same paragraph he observes, "The experiments of Dr. Philip show similar results; but there is one peculiar observation of his that is very interesting, which is, that when the action of the heart has been much disturbed by the destruction of a certain portion of the spinal marrow, by waiting a little time that action would resume its former regularity; and then another portion of the spinal marrow might be destroyed without stopping it; and so on repeatedly." At the end of this sentence there is a reference to my Inquiry, and even to the particular experiments,—experiments which I never made; as Mr. Hutchinson will find, if he ever takes the trouble to read my Inquiry. All this would be quite amusing, were not so gross a violation of the implied compact between a reviewer and the public too grave a subject for merriment.

As Mr. Hutchinson appears to be wholly unacquainted with the contents of my Inquiry, it is not surprising that, in page 22, he should argue against my supposed opinion, that the existence of some part of the spinal marrow is necessary to the action of the heart, although I have detailed many experiments in the very beginning of the Inquiry, for the purpose of proving that the power of the heart is wholly independent of every part of the spinal marrow, its action not being in the least influenced by the total removal of that organ. In the next paragraph he ascribes to me the opinion, that the ganglian system retains the nervous influence "like a sort of reservoir;" whereas I have made many experiments, and detailed many observations, expressly with a view to prove that the ganglian system is not a reservoir of nervous influence, but a mere channel through which it flows. But the reader has seen enough of Mr. Hutchinson's suppositions. I do not mean to say that he is intentionally incorrect; but the effect, as far as relates to the public, is the same as if he

---

* First edition, page 61; pages 240, 241, 242, 243. Second edition, page 61; pages 250, 251, 252, 253.

† Second edition, page 62.

were so, and such unaccountable carelessness in a man, who professes to judge others and direct public opinion, is little less culpable than intentional error.

Where there is such incorrectness in the statements, we expect to find equal inaccuracy in the reasoning. Mr. Hutchinson's reasoning does not disappoint this expectation.

### Mr. Hutchinson's *Reply to the Remarks of* Dr. Wilson Philip.

I solicit for a few minutes the attention of the reader of those statements of Dr. Wilson Philip that apply to me, that I may render it evident to him, notwithstanding the manner in which those statements are advanced, that, with the exception of two trivial points, they admit of the most decisive refutation.

In two instances I have erred in the statements referred to by Dr. Wilson Philip, the one, in saying that this gentleman had not noticed the acephalous fetus; the other, in attributing to him experiments which were made by Legallois. But I did not by this impugn any opinion of Dr. Wilson Philip; and those experiments, if applied to his opinions, must *by him* be considered to *favor* them: the facts were impressed on my mind, and adducing them amongst my arguments seven years after the perusal of the work of the French physiologist, and two after that of the latter gentleman, my memory so far deceived me as to make me attribute to the repeater of the greater part of Legallois' experiments what is due to Legallois himself. Although I daily have occasion to congratulate myself on my memory and powers of recollection, I cannot in this instance be surprised that they have deceived me; the principle, that the action of the heart will go on after the destruction of the spinal marrow, is the most important one established in Dr. Wilson Philip's 'Inquiry'; it does not, therefore, I repeat, surprise me, that the idea of that gentleman was identified with the executor of those experiments which most clearly prove the fact just alluded to.* This is an error, I confess it with regret; but the branding it with the epithet of a "*gross violation of the implied compact between a reviewer and the public*," as Dr. Wilson Philip has done, is a "*gross violation*" of propriety in that gentleman's manner of stating the case in question. It has no relation to the "compact between a reviewer and the public." I was not reviewing his book, but arguing on a point of Physiology, and referred to Dr. Wilson Philip's '*Inquiry*,' as I did to the works of Bichat, Legallois, Pfaff, and others, for such facts as were impressed on my mind, and which I thought proper to adduce.

Dr. Wilson Philip says, "What is meant by the assertion that I have refuted the notions of Bichat respecting the [*functions of the*, should come here. See *Proëmium*, page xx.] ganglian system and the loose and remote analogies, I know not." The best reply on my part to the first of these propositions is the following passage from the second edition of this gentleman's '*Inquiry*:' "It seems to be superfluous, after the experiments which have been related, to say any thing in refutation of the opinion of Bichat, that the ganglians are centres of nervous influence, independent of the brain and spinal marrow."—(*Inquiry*, page 185.) I have stated in the *Proëmium* (page xxiii.) some of

* The candid reader of this, who has also read the *Proëmium*, is entreated to reflect for an instant on the multitude of observations comprised in that production; the immense number of books, papers, journals, &c. that must have been perused in order to furnish the matter for it; that many of the books, &c. of which comprehensive accounts are given, were not published, or did not arrive in England until three or four weeks before the publication of the *Proëmium* itself; that it was written in one fortnight; and they will I think acknowledge, that the attributing, *in a corollary remark*, to one man an experiment made by another, *under the circumstances of the case in question*, and *where no person's opinion is impugned*, is a very trivial error, and a fit subject for amicable correction, not derision.

what I consider to be Dr. Wilson Philip's "loose and remote analogies;" extending the list of them, is not, I consider, necessary in order to substantiate my assertion.

Dr. Wilson Philip says, with a reprobative air, that I passed "unnoticed various other parts of my (Dr. Wilson Philip's) treatise, all the experiments, for example, which I have detailed for the purpose of proving, that the organs which are under the ganglian system, are affected by stimuli applied to the brain and spinal marrow." I passed those experiments unnoticed, because I did not think it necessary to notice them in the view I took of the physiological question on which I was reasoning;* and had I thought proper to notice such experiments, it is likely that I should have chosen what I consider more authentic sources in my reference to them. So far, however, from being unacquainted with the fact to which Dr. Wilson Philip alludes, I reason on it in the course of my arguments on the question alluded to.—(See *Proëmium,* page xxiii.)

Dr. Wilson Philip says also, I argue against his "supposed opinion that the existence of some part of the spinal marrow is necessary to the action of the heart." Here my statement is placed in a wrong aspect. I began by saying that the only important argument brought against the doctrine of Bichat is that "THE FUNCTIONS HE STATED TO DEPEND ON THE INFLUENCE OF THE GANGLIONIC SYSTEM OF NERVES, *cease on the destruction of the brain and spinal marrow;*" and though, in page xxii. I use the expression *action of the heart,* as a mean of designating the existence of those functions, I refer to a note in which I expressly state this.† I use the same term in the same sense in page xxi.; and there, too, I refer to a note, stating that Haller's doctrine of this, which Dr. Wilson Philip has *adopted,* need not be discussed, because it is now generally abandoned. Dr. Wilson Philip acknowledges that the action of the heart will proceed after the destruction of the brain and spinal marrow, but he denies that "*the functions* (BICHAT) *stated to depend on the influence of the ganglionic system of nerves,*" will proceed after the destruction of the brain and spinal marrow. It was against this doctrine that I argued, and I believe my meaning has been perfectly intelligible to every other reader.‡

* The reader will bear in mind, that the second edition of Dr. Wilson Philip's '*Inquiry*' was published nearly a year previously to the period which I professed to comprise in my '*History,*' &c.; and therefore it was not a subject for regular analysis, though I might think proper to notice any particular parts of it, as I would do in a work published centuries ago. Every author whose opinions are noticed in the *Proëmium* might with equal propriety impute blame to me for not having adduced all the observations contained in his works.

† The note in question is "this phenomenon (*the action of the heart,* stated in the text), is alone mentioned, because it is the most sensible and important one; but the secretions and other functions of nutritive life, are in general similarly influenced by the experiments under consideration."

‡ As all the readers of this may not have seen the *Proëmium,* I shall transcribe here from it, what relates expressly to this part of the question. "The only important argument brought against the doctrine of BICHAT is, that the functions he stated to depend on the influence of the ganglionic system of nerves, cease on the destruction of the brain and spinal marrow.* It appears from the experiments of LEGALLOIS, as well as of DR. PHILIP, that it was only the *sudden* and *simultaneous* destruction of those parts of the nervous system that caused the death of the animal; for, if it were affected gradually, leaving an interval between the destruction of different portions, nearly the whole of it could be removed without producing death of the organic system:† and, what is of great importance in the consideration of this subject, Legallois found that the younger the animal was, the more freely its spinal marrow might be destroyed,‡ without causing cessation

* Let it be borne in mind that those functions are proved to go on without the existence of the brain and spinal marrow in the fetuses above referred to.

† That is, provided the lungs were artificially dilated with air at proper intervals. The dilatation of the cavity of the chest depends on the action of muscles subservient to the influence of the brain and spinal marrow, though the changes of the blood in the lungs depend on the ganglionic system.

‡ It is worthy of particular remark, that the action of the heart was less severely affected by the destruction of the spinal marrow after the head and brain had been separated from the body, than when this remained attached to it.

Dr. Wilson Philip's next remark is, "he ascribes to me the opinion, that the ganglian system retains the nervous influence, 'like a sort of reservoir;' whereas I have made many experiments, and detailed many observations expressly with a view to prove, that the ganglion system is not a reservoir of nervous influence, but a mere channel through which it flows." The best reply to this, is a passage in the second edition of that gentleman's '*Inquiry*:' but I will first transcribe my own remark just alluded to.

"Another supposition that Dr. Philip has adopted to support his hypothesis, is, that the ganglionic system derives its nervous influence from the spinal marrow, but that it retains it for some time, like a sort of reservoir; and therefore, the actions dependant on its immediate influence, proceed until this supply is exhausted."—(*Proëmium*, p. xxii.)

"Although we have reason to believe, I think, from every observation on the subject, that the brain and spinal marrow are the only sources of nervous influence; yet it is evident that a certain portion of this influence remains in the nerves when separated from these organs, as appears from the contractions excited in the muscles by irritating their nerves under such circumstances. The muscle will thus be made to contract as long as any influence remains in the nerve, but this being once exhausted, the nerve has no means of renewing it." *Inquiry*, p. 203 and p. 204, it is stated, "as long as we can, by artificial respiration, occasion such a change in the blood as elicits nervous influence, the blood draws it from all the nerves of the ganglian system; and it does not appear that we can support this change long enough to exhaust the nervous influence already in the nerves, and occasion any further demand for it."

Dr. Wilson Philip has not used the term *reservoir*, but he has stated what must convey the same *idea*. The functions in question (some of my readers may perhaps not know it), will go on for several hours, under the circumstances above designated. If, then, the ganglionic nerves continue to supply nervous influence during that time, when the sources of such influence are removed, must they not retain it "like a sort of reservoir"? I know no epithet in the English language better calculated to express in one term the substance of the many sentences in which Dr. Wilson Philip has developed the same idea as that epi-

---

of the circulation and the changes of the blood in the capillaries from arterious and venous. Before the age of ten days, the only constant phenomenon was "the sudden cessation of the circulation on the *simultaneous destruction of the whole* of the spinal marrow.* It was only weakened or disturbed more or less by the destruction of *any one* of the three portions into which he distributed it, that is, the thoracic, dorsal, and lumbar; though the destruction of any one of those portions in a rabbit a few days older would cause the action of the heart to cease. He also found that, by making the destruction at five or six different times, the greater part might be destroyed, and yet the circulation would proceed.† The experiments of Dr. Philip show similar results. But there is one peculiar observation of his that is very interesting; which is, that, when the action of the heart has been much disturbed by the destruction of a certain portion of the spinal marrow, by waiting a little time, that action would resume its former regularity; and then another portion of the spinal marrow might be destroyed without stopping it, and so on repeatedly.‡

If, then, any certain portion of the spinal marrow, and nearly all of it, may be destroyed without the cessation of the action of the heart,§ what is the conclusion that should be thence formed, but that the existence of the spinal marrow is not essentially necessary for the action of the heart? Dr. Philip, however, says, that any part of the ganglione system may obtain its nervous energy from any part of the spinal marrow: thus the nerves supplying the heart may derive it from the lowest part of the spine.

* *Experiences sur le principe de la vie*, page 97.

† It is not necessary to say any thing in refutation of Haller's explanation of this. His doctrine is now generally abandoned.

‡ *On the laws of the vital functions.* Exp. 19, 20, 21, 22.

§ This phenomenon is alone mentioned, because it is the most sensible and important one; but the secretions and other functions of nutritive life, are in general similarly influenced by the experiments under consideration.

that conveys. Here, then, my supposition of that gentleman's opinion is merely a repetition *in other words*, but *not in other ideas*, of *his own statements*; of *which*, I agree with him, "*I think the reader has seen enough*." To his remark on my reasonings, I only reply, that I am not at all desirous that he should entertain a more favourable opinion of them; nevertheless, I hope they are correct; but if not so, I hope with equal earnestness that some of my readers will point out in what respect I have erred in them; and, I am confident that there are but very few of them who will not consider that the zeal in the cause of medical science manifested in the London Medical and Physical Journal under its present superintendance, is sufficient evidence to show that I shall receive precise statements of my errors with as much zeal as I endeavour to propagate truths. I should feel honoured by any of them who would open a liberal controversy on the physiological points above alluded to; and, lest the marks of inurbanity which have been just manifested should continue to excite their disgust, I must remark, that it is I who have been the subject of them; on no other occasion would they have appeared in this Journal: and I hope I shall be pardoned for having turned back the arrows impelled against me on this occasion. W. HUTCHINSON.

*Sackville Street, Feb.* 24, 1820.

